# Vascularized skin tissue models featuring adipose cell spheroid-laden GelMA hydrogels

**DOI:** 10.1016/j.mtbio.2025.101835

**Published:** 2025-05-05

**Authors:** Dongjin Lee, Sangmin Lee, Jeongbok Lee, Dahong Kim, Hyunseok Kwon, Junhyoung Ahn, Hyungjun Lim, Jae Jong Lee, Heungsoo Shin, Su A Park

**Affiliations:** a Nano-Convergence Manufacturing Research Division, Korea Institute of Machinery and Materials (KIMM), Daejeon, 34103, Republic of Korea; b Department of Bioengineering, Hanyang University, Seoul, 04763, Republic of Korea; c BK21 FOUR, Education and Research Group for Biopharmaceutical Innovation, Hanyang University, Seoul, 04763, Republic of Korea; d Department of Applied Bioengineering, Graduate School of Convergence Science and Technology, Seoul National University, Seoul, 08826, Republic of Korea; e Institute of Nano Science and Technology, Hanyang University, Seoul, 04763, Republic of Korea

**Keywords:** 3D bioprinting, 3D scaffold, Skin-adipose complex tissue, Hydrogel stiffness, Adipose spheroids

## Abstract

The multifaceted tissue interplay between skin and adipose structures is increasingly recognized to play crucial roles in antimicrobial defense, hair cycling, wound healing, and thermogenesis. However, the technical challenges associated with the development of an *in vitro* model of such complex tissues include the difficulties of integrating tissues with diverse characteristics. Here, we present a method using a gelatin methacryloyl (GelMA) hydrogel to establish a microenvironment that hosts connected composite tissues: a vascularized skin layer and a subcutaneous adipose layer. When adipogenesis proceeded in 3T3-L1 cell spheroid-laden three-dimensional (3D)-printed polycaprolactone (PCL) scaffolds after 1- and 2-min exposure to ultraviolet (UV) light, we observed that adipose tissue, the physical properties of which had been optimized by 1-min UV exposure, facilitated the migration and proliferation of 3T3-L1 cells. Furthermore, a notable enhancement in adipogenesis was apparent. Subsequently, using advanced 3D printing technology, we meticulously crafted a 3D vascularized skin layer by integrating microgels with human umbilical vein endothelial cells (HUVECs) and fibroblasts. HUVEC cells growing on the surface of the microgel exhibited a 3D structure that allowed vascular cells to become concentrated in the microgel area much more efficiently than in 2D culture. Three-dimensional printing allows efficient mass production, removing challenges that cannot be easily addressed via *in vivo* experiments. In the immediate future, we will simulate complex pathological conditions such as burns, psoriasis, and atopy. Our approach will facilitate the discovery of useful treatments for these conditions.

## Graphical abstract

**Figure:**
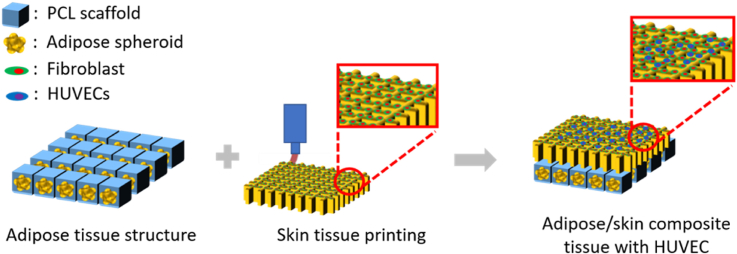

## 1 Introduction

Cutaneous tissue functions as the primary barrier against an often hazardous external milieu, serving as a protective integument for the body. Subcutaneously, the reticular dermis harbors a dermal adipose tissue layer that serves as an energy reservoir, engages in thermal regulation, and provides structural support [1]. Recent studies have highlighted the intricate interplay between skin and adipose tissues, with an emphasis on their pivotal roles in antimicrobial defense, hair cycling, wound healing, and thermogenesis [2]. Despite increasing awareness of the major activities performed by the intricate architectures of skin and adipose tissue, the development of an *in vitro* model replicating this composite structure, particularly a dual-layered model, is technically challenging. Animal experiments have traditionally been used to explore the biological mechanisms. However, recently, the emphasis has shifted toward *in vitro* models, prompted by the recognition of erroneous conclusions stemming from the disparities between non-human organisms and humans [3]. Although extensive research has been conducted using *in vitro* models, conventional two-dimensional (2D) models are constrained by difficulties in faithful replication of the complex native tissue, attributable to variation in the composition of tissue-specific cell types and the extracellular matrix (ECM) and differences in how cells respond to external stimuli, often influenced by the size of the exposed cell surface area [4]. As a result, the currently preferred approach is to apply three-dimensional (3D) biofabrication technologies to emulate the hierarchical microenvironment, enhancing the similarity to the *in vivo* milieu [5]. A recent study fabricated PMSA hydrogel small-diameter vascular grafts with excellent biocompatibility and mechanical properties using an economical and controllable 3D mold, and demonstrated excellent patency in an animal model [6]. Most studies have conducted vascularization studies by simply mixing or seeding two-dimensional HUVEC cells with biopolymers [7]. However, our study, unlike the existing method, attempted to mimic a three-dimensional form similar to the biological environment by coating the HUVEC cells on the surface of the microgel, culturing them, and transplanting them.

To mirror the characteristics of authentic skin tissue, one basic approach involves the development of a full-thickness skin model [8]. The epidermal layer is mimicked via air-liquid interface culture of keratinocytes, and the dermal layer (or dermis) is replicated by encapsulating fibroblasts in a hydrogel [9]. Several authors also have incorporated melanocytes and/or endothelial cells into such structures, or have used rheological methods to replicate the pigmentation, vascularization, and wrinkles of real skin tissue [10,11]. Likewise, adipose tissue models employing 2D cultures of adipose cells, including immature and/or mature adipocytes, adipose-derived stem cells (ADSCs), and endothelial cells, do not accurately represent the intricate complexity of adipose tissue [12,13]. Among the available adipogenic cell models, the murine pre-adipocyte cell line 3T3-L1 has been widely adopted due to its well-established adipogenic differentiation capacity, consistent lipid accumulation, reproducibility across experiments, and practical advantages in culture and genetic manipulation [14]. Hence, recent research has focused on the dynamic 3D culture cell spheroids formed via self-assembly of cell-laden 3D hydrogels; these accurately replicate the 3D microenvironment [15,16]. Specifically, spheroids enhance ECM expression and adipogenic differentiation by maximizing cell–cell interactions, facilitating the creation of more adipose tissue-like specific microenvironments [[17], [18], [19]]. Moreover, volumetric constructs prepared using hydrogels emulate the volumetric filling characteristics of adipose tissue [20]. When reconstruction must be comprehensive, biofabrication necessitates the inclusion of skin cells, the specific vascular cells of each layer, and adipose cells. This establishes an integrated microenvironment that mirrors the complex tissue construct that encompasses both skin and adipose tissues.

Three-dimensional bioprinting technology is rapidly evolving to simulate complex 3D biological structures that cannot be realized in two dimensions; living cells and biomaterials are employed during layer-by layer-printing. As the manufacturing process is additive in nature, bespoke tissue constructs can be generated with high flexibility and reproducibility [21,22]. Pressure-based bioprinting, specifically extrusion bioprinting, is the predominant form of skin tissue engineering, facilitating the printing of a diverse range of biomaterials with varying viscosities at room temperature. Commonly, pneumatic pressure or mechanical pistons are used to deposit biomaterials [[23], [24], [25]]. Various types of skin cells, including melanocytes, endothelial cells, pericytes, microvascular endothelial cells, and stem cells from diverse sources, have been incorporated into 3D-bioprinted skin constructs [26]. Among the various natural materials, gelatin, a well-known biological substance, is extensively used given its excellent biocompatibility, biodegradability, and lack of toxicity. Gelatin, derived via denaturation and hydrolysis of collagen, the primary component of the native ECM, exhibits several distinct advantages. However, its poor mechanical properties and the lack of stability within aqueous and physiological environments render gelatin unsuitable for medical applications [27]. The various gelatin crosslinking methods are physical, chemical, or enzymatic in nature [28]. To address the limitations described above, we introduced double bonds into gelatin when creating a gelatin methacryloyl (GelMA) polymer that could undergo ultraviolet (UV) light-mediated cross-linking in supporting information (Fig. S1). Many studies have been introduced using biopolymer GelMA [29]. A recent study simulated the effect of human bone marrow adipocytes on prostate cancer bone metastasis using a GelMA hydrogel-based 3D platform. In addition, a new therapeutic strategy was proposed to promote wound healing and vascular regeneration of aged skin using GelMA hydrogel containing hypoxic pretreated ADSCs [30].

**Fig. 1:**
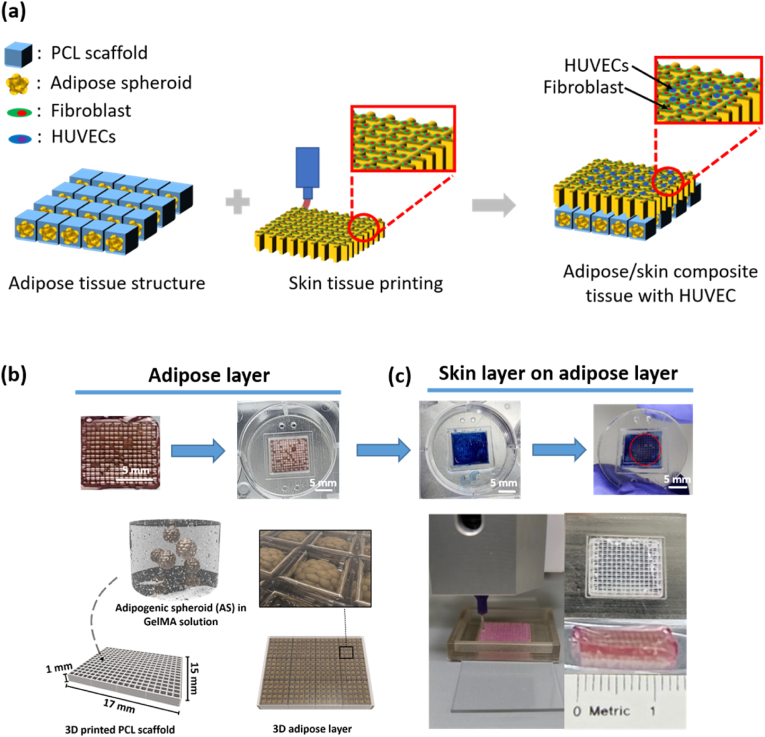
(a) Schematic of the adipose/skin complex tissue created via 3D bioprinting employing HUVECs, fibroblasts, and 3T3-L1 cells. (b) Spheroids embedded in the PCL scaffold formed a 3D adipose layer. (c) Three-dimensional bioprinting onto the adipogenic tissue layer using GelMA bioink, with inclusion of fibroblasts and HUVECs to mimic a complex tissue composed of adipose tissue and skin.

In this study, we created a sophisticated dual-layer tissue model with vascularized skin and adipose layers. Initially, we meticulously prepared size-adjustable pre-adipocyte spheroids of 3T3-L1 cells and incorporated them into a 3D-printed polycaprolactone (PCL) scaffold having a GelMA hydrogel with precisely tuned mechanical properties. Subsequent analyses focused on the migration, proliferation, and adipogenesis of 3T3-L1 cells within this bespoke microenvironment. To ensure the fidelity of the adipose and skin layers, microgels were strategically employed as 3D structural elements that facilitated vascularization by green fluorescence protein-labeled human umbilical vein endothelial cells (GFP-HUVECs). The biocompatible polymer GelMA was chosen for construction of adipose/skin complex tissue. In the initial layer, adipose spheroids were strategically positioned on the GelMA hydrogel atop a PCL scaffold, followed by the sequential creation of a skin layer via 3D printing directly onto the adipose tissue, as illustrated in Fig. 1a. The overall dimensions of the composite construct are 1.4 cm in width, 1 cm in height, and 2 mm in depth.

## 2 Materials and methods

PCL (molecular weight [mW], 45,000 Da) was purchased from Sigma-Aldrich (St. Louis, MO, USA). Dexamethasone(D4902), IBMX(I5879) and indomethacin(I7378) were also from Sigma-Aldrich. Distilled water (DW) was obtained from Elix Advantage (Millipore, MA, USA). Phosphate-buffered saline (PBS), Dulbecco's phosphate-buffered saline (DPBS), and trypsin/ethylenediaminetetraacetic acid (EDTA, LS015-01) were purchased from Welgene (Gyeongsan-si, Korea). Dulbecco's modified Eagle's medium (DMEM, DME001) was from Solbio (Seoul, Korea). 3T3-L1 cells were acquired from the Korean Cell Line Bank (Seoul, Korea). Fetal bovine serum (FBS, 12483020), bovine calf serum (BCS, 16170078), and a penicillin/streptomycin (PS, 15140122) mixture were obtained from Gibco (Waltham, MA, USA). Oil red O (1320-06-5) and BODIPY™ 505/515 (D3921) were purchased from Sigma and Invitrogen (Carlsbad, CA, USA) respectively. A mounting medium that included 4′,6-diamidino-2-phenylindole (DAPI, h-1200) was purchased as VectaShield® (Vector Laboratories, Burlingame, CA, USA). GelMA was acquired from 3D Materials (20BT27, Busan, Korea); GelMA was synthesized from gelatin type A of porcine skin and the extent of methacrylation was approximately 90 % (according to the manufacturer's data). A UV spot-curing system was purchased from UV SMT (Bucheon, Korea). Calcein-AM(4781107) and rhodamine phalloidin(R415) were from Invitrogen. An RNeasy Mini Kit(74104) was purchased from Qiagen (Valencia, CA, USA). Maxime RT Premix was obtained from Intron (Seoul, Korea), and the SYBR Premix Ex Taq(RR420A) was purchased from TAKARA (Shiga, Japan). HUVECs were obtained from Lonza (Basel, Switzerland), and fibroblast (NIH-3T3) cells were from ATCC (Manassas, VA, USA).

### 2.1 Fabrication of the PCL scaffold (a basket structure)

A PCL scaffold was fabricated using an extrusion-based printing approach employing a 3D bioprinter (constructed in-house at the Korea Institute of Machinery and Materials) [[31], [32], [33]]. The instrument and the fabrication process were as described previously [[34], [35], [36], [37], [38]]. Briefly, the 3D printing system featured a heating jacket, a 200-μm-diameter nozzle, a pressure pump with an x-y-z stage, and computer software. PCL pellets were melted in a heating cylinder at 100 °C for 30 min. The basket strand sizes and between-strand distances were varied using a computer-aided design (CAD) model followed by computer-aided manufacturing (CAM). This allowed layer-by-layer printing of a complex, interconnected 3D structure. The strand thickness and between-strand distance were 200 and 700 μm, respectively. The printing speed was 300 mm min^−1^, and the pneumatic pressure was 420 kPa. The strand of the PCL is described in Supporting Information (Fig. S2).

### 2.2 Characterization of the PCL scaffold with a GelMA hydrogel at the base of the adipose layer

The 3D-printed PCL scaffolds were dispersed in a dopamine hydrochloride solution (2 mg/mL in 10 mM Tris-HCl buffer, pH 8.5) at a concentration of 2 mg/mL. Subsequently, the samples were incubated at 37 °C with gentle agitation (50 rpm) for 30 min and washed twice with DW. Polydopamine coatings on the scaffolds were quantified using the micro-bicinchoninic acid (BCA) assay. Samples (*n* = 4) were cut into 7.0 cm^2^ sections and incubated with 100 μ L of micro-BCA working solution (Pierce, Rockford, IL, USA) at 37 °C for 2 h. Absorbance was measured at 562 nm using a microplate reader (Varioskan LUX, Thermo Scientific, Waltham, MA, USA). For release studies, the coated scaffolds were placed in 6-well plates and immersed in 2 mL of DW. At days 1, 4, and 7, the entire volume was collected and replaced with fresh DW. The amount of released catecholamine was quantified using the BCA assay, and results were normalized to the initial coating amount (defined as 100 %). The prepared PCL and PD-coated PCL scaffolds were then dried, and their surface morphologies were examined using scanning electron microscopy (SEM) (Hitachi S-4800; Hitachi, Ltd, Tokyo, Japan). The surface functional groups on both PCL alone and PD-coated PCL scaffolds were analyzed using attenuated total reflection-Fourier transform infrared spectroscopy (ATR-FTIR) (Nicolet 6700; Thermo Scientific, Waltham, MA, USA). X-ray photoelectron spectrometry (XPS) was performed to characterize the surface composition of both PCL and PD-coated PCL scaffolds, using a Theta Probe Base System (Thermo Fisher Scientific). The acquired spectra were processed with Thermo VG Scientific software (MA, USA). The GelMA solution (7.5 % w/v in DPBS) was filtered through a syringe filter (0.2-μm pore diameter) and mixed with 2-hydroxy-2-methylpropiophenone (0.1 % v/v). PD-coated PCL was then positioned in a custom-made polytetrafluoroethylene (PTFE) mold (3 cm in width × 2.4 cm in height × 1 mm in depth) and soaked in the prepared GelMA solution (400 μL). The scaffold-laden GelMA solution was then crosslinked under UV (350 mW) for 1 or 2 min. The storage moduli of the scaffold-laden hydrogels after 1 or 2 min of UV exposure were measured using a rheometer (HR10; TA Instruments, New Castle, DE, USA) under an axial force of 0.4 N and 1 % strain.

### 2.3 Characterization of 3T3-L1 spheroids embedded in PCL scaffolds with the GelMA hydrogel

3T3-L1 cells were cultured in DMEM (with 1 % PS and 10 % BCS, both v/v) under standard conditions (5 % v/v CO_2_ and 37 °C) to a confluency of <80 % prior to expansion, with fewer than eight passages for all experiments. In a 96-well cell floater plate (SPL Life Sciences, Gyeonggi-do, Korea), 4 × 10^4^ or 8 × 10^4^ 3T3-L1 cells were centrifuged at 1200 rpm for 5 min and then incubated for 24 h to form spheroids. Spheroid shapes and sizes were monitored using phase-contrast microscopy and analyzed with Image-Pro Plus software (Media Cybernetics, Silver Spring, MD, USA). Additionally, 3T3-L1 spheroids with 4 × 10^4^ or 8 × 10^4^ cells were fixed in 4 % v/v paraformaldehyde, embedded in optimal cutting temperature (OCT) compound, and frozen at −80 °C to yield OCT blocks. The blocks were sectioned into 8-μm-thick units using a cryostat microtome, and the cross-sectioned spheroids were incubated in DW for 10 min, stained with hematoxylin for 2 min and eosin for 8 min, and preserved using a mounting medium prior to observation. 3T3-L1 spheroids (with 4 × 10^4^ or 8 × 10^4^ cells) were mixed with GelMA solution, sprinkled onto PD-coated PCL scaffolds, and crosslinked under UV light for 1 min. Spheroids of 8 × 10^4^ 3T3-L1 cells with PD-coated PCL scaffolds were crosslinked via UV exposure for 1 or 2 min and placed in a six-well cell floater plate (SPL Life Sciences) with 3 mL of growth medium/well that was replaced every 2 days for 14 days. The constructs were incubated in calcein-AM solution for 20 min at 37 °C, and the sprouting morphologies of 3T3-L1 cells (from spheroids to GelMA hydrogels) were observed using a fluorescence microscope. To measure DNA content, spheroid-laden PCL scaffolds with hydrogels cultured for 1, 7, and 14 days were incubated in collagenase solution (100 units in PBS) and centrifuged at 14,000 rpm for 2 min. The isolated spheroids were homogenized in 1- mL amounts of RIPA buffer, and the DNA contents of 3T3-L1 cells were analyzed using the Quant-iT PicoGreen dsDNA assay kit (Invitrogen).

### 2.4 Adipogenesis in 3T3-L1 spheroid-laden PCL scaffolds with the GelMA hydrogel

3T3-L1 spheroid-laden PCL scaffolds with GelMA hydrogels were cultured for 1–7 days in general medium and then in adipogenic differentiation medium (DM) (DMEM with 1 % PS and 10 % FBS [both v/v] containing 2.5 μM dexamethasone, 1.25 mM IBMX, and 250 μM indomethacin) for 7–14 days. Constructs cultured for 14 days were fixed with 4 % (v/v) paraformaldehyde and incubated with BODIPY™ 505/515 or oil red O solution. Incubated with rhodamine phalloidin solution for 1 h at 37 °C, diluted 1:100 in DPBS, washed three times with DPBS, and incubated for 1 h with BODIPY™ 505/515 staining solution (3.8 μM in DPBS). After two washes with DPBS, samples were preserved in a mounting medium that contained DAPI prior to observation using a confocal laser microscope. Similarly, for oil red O staining, the constructs were washed with DW four times and treated with 100 % IPA for 10 min, followed by incubation with the oil red O working solution (the filtered 60 % [w/v] stock solution in DW; the stock solution was 3 % [w/v] oil red O in IPA) for 30 min at 37 °C. After washing four times with DW, oil red O-stained samples were preserved using mounting medium and examined using a phase-contrast microscope. Each oil red O-stained area (%) normalized to the total area was measured via image analysis using Image-Pro Plus software. Constructs stained with BODIPY™ 505/515 were observed using a confocal microscope, and the diameters of the lipid droplets, which fluoresced green, were measured via image analysis. To isolate 3T3-L1 spheroids, constructs cultured for 14 days were chopped, incubated in collagenase solution (100 units in PBS), and centrifuged at 14,000 rpm for 2 min. Subsequently, the isolated 3T3-L1 spheroids were lysed using RLT buffer. The mRNAs from 3T3-L1 cells within the spheroids were extracted using an RNA extraction kit (Qiagen, Hilden, Germany). The total RNA concentrations were measured with a nano-spectrometer (Nanodrop 2000; Thermo Scientific, Wilmington, DE, USA), and cDNA was then synthesized using a Maxtime RT PreMix Kit. The cDNAs were mixed with SYBR Green PCR MasterMix, and real-time reverse transcription polymerase chain reaction (RT-PCR) was performed using a StepOnePlus thermocycler (Life Technology, Carlsbad, CA, USA). The primers used for PCR had the following sequences: C/EBPA (Fw: 5′-CGG TGG ACA AGA ACA GCA AC-3′, Rv: 5′-CGG AAT CTC CTA GTC CTG GC-3′); PPARG (Fw: 5′-TGT CTC ATA ATG CCA TCA GGT TTG-3′, Rv: 5′-GAT AAC GAA TGG TGA TTT GTC TGT T-3′); FABP4 (Fw: 5′-ACC AGG AAA GTG GCT GGC AT-3′, Rv: 5′-CAG GTC AAC GTC CCT TGG CT-3′); adiponectin (Fw: 5′-AGC CTC CTT CTC CTG GGT CC-3′, Rv: 5′- GTT GCC TCT AGC CTG GTG GG-3′).

### 2.5 Analysis of GelMA physical properties

We used GelMA Bioink (7 % w/v) to measure extrudability according to the printing head temperature (5–37 °C) and nozzle diameter (100–500 μm) while maintaining the pneumatic pressure at 100 kPa. The rheological behaviors of GelMA hydrogels (6 %, 7 %, and 8 % w/v) were examined using a rheometer (AR2000ex; TA Instruments, TX, USA) with a 40-mm aluminum plate held at 15°C. The samples were placed on a bottom plate, and a parallel plate was then fitted.

### 2.6 Cell culture

NIH3T3 fibroblasts (ATCC) were cultured in high-glucose DMEM (Capricorn; Hesse, DE, USA) supplemented with 10 % FBS and 1 % PS (both v/v) (Capricorn) at 37 °C under 5 % (v/v) CO_2_. The medium was replaced every 2 days. HUVECs (ATCC) were cultured as described previously [39]. Briefly, HUVECs were cultured in an EGM™-2 Endothelial Cell Growth Medium-2 BulletKit (EGM-2 CC-3162; Lonza) containing SingleQuots™ Supplements (Lonza), such as FBS, at 37 °C in a humidified atmosphere of 5 % (v/v) CO_2_. Subsequently, the cells were grown to 80 % confluence and subcultured after detachment using 0.05 % (w/v) trypsin-EDTA. HUVECs at passage <7 were used in this research.

### 2.7 Characterization of skin layers prepared using various GelMA concentrations with NIH3T3 fibroblasts and co-cultured with HUVECs

To ensure that fibroblasts in the skin layer grew well in the GelMA environment, the cells were cultured for 1 week at various GelMA concentrations ranging from 6.5 % to 7.5 % w/v. The Premix WST-1 Cell Proliferation Assay System (TAKARA) was used to measure mitochondrial dehydrogenase activity during proliferation of NIH3T3 fibroblast cells in GelMA. The key reagent was prepared by mixing the WST-1 solution and the basal culture medium (DMEM) at a volume ratio of 10:1, and absorbance was then measured using a microplate reader (SpectraMax iD3; Molecular Devices, CA, USA). In addition, GelMA solutions at various concentrations (6.5 %–7.5 % w/v) in PBS (pH 7.4), with 0.2 % (w/v) Irgacure2959 as a photoinitiator, were prepared. Each GelMA solution was placed in a glass mold 1 mm in height and UV-irradiated for 2 min (intensity: 265 mW, emission filter: 250–450 nm, distance: 3 cm; Model S1500; Omnicure, MA, USA) to fabricate GelMA disks that were then punched out (8 mm in diameter). These GelMA disks were held in PBS for 24 h before measurement of mechanical properties via derivation of elastic moduli using uniaxial compression tests (Model 3343; Instron; MA, USA) in Supporting Information (Fig. S3) [40,41]. GelMA bioink was gently mixed with NIH3T3 fibroblasts and HUVECs in a 1:1 ratio (5 × 10^4^) followed by culture for up to 2 days in a 12-well plate.

### 2.8 Fabrication of microgels

The channel geometry of the microfluidic device is described in Supporting Information (Fig. S4). The concentrations of GelMA in the primary (Aq1) and secondary (Aq2) aqueous phases were 9 % and 12 % (w/v) [42]. Both Aq1 and Aq2 included 0.2 % (w/v) Irgacure2959 as a photo-initiator. Mineral oil and 20 % (v/v) Span80 (Sigma Aldrich) were mixed to form the oil phase. The fluids were injected into the microfluidic chip at a constant rate using electronic syringe pumps (Legato100; KD Scientific, MA, USA). To ensure that the generated droplets were of uniform size, the flow rate of Aq1 and Aq2 was set to 140 μL h^−1^, and the flow rate of O was 900 μL h^−1^. The droplets were exposed to UV light for 2 min (intensity: 265 mW, distance: 3 cm, emission filter: 250–450 nm; Model S1500; Omnicure); this crosslinked the droplets into microgels that were washed with PBS three times to remove residual oil from the microgel surfaces.

### 2.9 Construction of a skin layer with fibroblasts and 3D microgel-coated GFP HUVECs

To attach GFP-HUVECs to the microgel surface, pre-fabricated microgels were first placed in an uncoated 12-well plate, and GFP-HUVECs were then seeded into the plate followed by culture for up to 3 days. To check whether GFP-HUVECS coated on the microgel spread to other areas of the GelMA, seeded cells were observed on the GelMA surface.

### 2.10 Skin layer on adipose tissue

To distinguish between fibroblasts in the skin layer and HUVECs expressing GFP, medium-free fibroblasts were exposed to the Cell Tracker™ CM-Dil (Red) reagent for 30 min. The cells were stained red and washed three times with PBS. After staining, fluorescent images of cells within microgels were visualized using a confocal microscope (FV1000; Olympus, Tokyo, Japan).

### 2.11 Fabrication of dual layers: skin layer and adipose layer

The skin layer was printed on the adipose layer fabricated via 1- or 2-min UV exposure. Dual-layer constructs cultured for 5 or 10 days were fixed with 4 % (v/v) paraformaldehyde and then treated with a blocking buffer with 0.1 % (v/v) Tween-20 and 5 % (v/v) FBS in PBS for 24 h at 4 °C. Subsequently, the samples were incubated for 24 h at 4 °C with a rabbit anti-CD31 antibody diluted 1:100 in blocking buffer. Next, the samples were sequentially incubated for 2 h at 37 °C with goat anti-rabbit IgG H&L (Alexa Fluor® 647) diluted 1:200 in blocking buffer, and then with a BODIPY™ 505/515 staining solution and a rhodamine phalloidin solution as described above. Finally, the samples were preserved in mounting medium containing DAPI for later observation under a confocal laser microscope. Samples cultured for 5 or 10 days and then stained for immunofluorescence were vertically cut, and the cross-sectioned samples were observed under a confocal microscope.

### 2.12 Statistical analysis

The results are expressed as means with standard deviations (SDs). Significance was determined using two-way ANOVA. Asterisks in figures denote significance at p < 0.05. All statistical analyses were conducted using Origin software (version 8.6; OriginLab Corporation, Northampton, MA, USA).

## 3 Results and discussion

### 3.1 Fabrication of a complex of skin and adipose tissue

We created a complex adipose/skin tissue using GelMA as the biocompatible polymer. In the first layer, fat spheroids were supported by a GelMA hydrogel in a PCL scaffold, and a skin layer was then created via 3D printing onto the adipose tissue in (Fig. 1a). Specifically, rather than using adipocytes to simulate a fat layer, we thought that cell differentiation into fat cells would be more appropriate; fat spheroids were thus cultured and introduced into the fat layer in (Fig. 1b). To simulate a composite fat/skin tissue, a skin layer was created on top of the prepared fat layer using NIH3T3 fibroblasts (representative skin cells). After co-culture with vascular cells, the skin layer was more closely simulated than its appearance might suggest in (Fig. 1c). As GelMA is transparent, it was difficult to distinguish the fat and the skin layers; thus, a blue dye was mixed with the bioink GelMA when printing the skin layer. After printing, spheroids in the fat layer appeared as red circles.

### 3.2 Characterization of PCL scaffolds with the GelMA hydrogel in the adipose layer

**Fig. 2:**
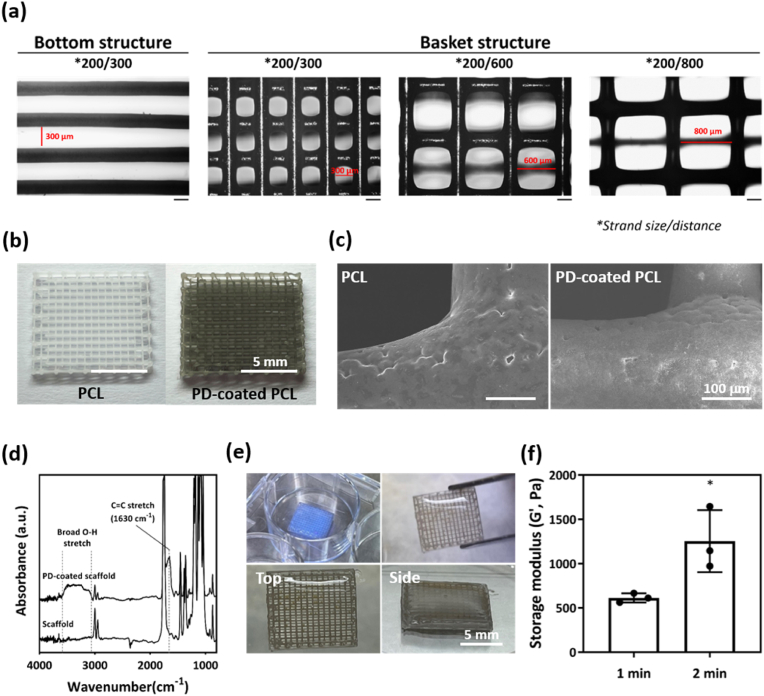
Characterization of a PCL scaffold with the GelMA hydrogel used to create an artificial fat tissue. (a) Printability assessments of PCL scaffolds with different strand sizes and between-strand distances; various basket structures were possible. (b) Pre- and post-coating images of PCL scaffolds with polydopamine and (c) SEM images. (d) FT-IR spectra. (e) Cross-linking of the GelMA hydrogel to the PCL scaffold. (f) Storage moduli of GelMA after 1 or 2 min of UV exposure (*n* = 3, samples) (∗p < 0.05).

The printability assessments of PCL scaffolds that differed in basket strand sizes and between-strand distances are shown in (Fig. 2a). When a PCL scaffold was printed in white, a color change to black was observed after PD coating (Fig. 2b). The PD-coated PCL exhibited an amine content of 140.64 ± 11.86 μ g/cm^2^ and showed a cumulative detachment of 10.70 ± 0.10 % over 7 days (Fig. S5a and b). Although the surface of the PCL structure appeared to be smooth, small aggregates were observed on PD-coated PCL (Fig. 2c). ATR-FTIR revealed broad O-H stretching at 3000–3700 cm^−1^ and phenolic C=C stretching at 1630 cm^−1^ after PD coating (Fig. 2d). In the full XPS spectra, an N1s peak appeared on the surface of the PCL scaffold after PD coating, indicating the presence of nitrogen-containing groups (Fig. S6a). In the high-resolution C1s spectra, the PCL scaffold showed peaks corresponding to C=O (288.6 eV), C–O (286.0 eV), and C–H (284.5 eV), whereas the PD-coated PCL additionally exhibited a C–N peak at 285.4 eV (Fig. S6b). A schematic of the GelMA hydrogel pouring onto and then cross-linking on the PCL scaffold to form the required structure is depicted in (Fig. 2e). A structure crosslinked for 2 min under UV light (1254.33 ± 350.05 Pa) exhibited a significantly greater storage modulus than that of a structure crosslinked for 1 min (612.67 ± 51.52 Pa) (Fig. 2f) because of aggregate formation on the surface of the PCL scaffold as PD coating proceeded, followed by a color change. It is known that PD-coating of biomaterial surfaces is driven by dopamine oxidation at alkaline pH values, triggering formation of dopamine–quinone and 5,6-dihydroxyindole, the levels of which depend on the PD coating time [43]. Surface aggregates on PD-coated PCL scaffolds grew as the coating time was extended and the PD level rose [44]. Brief PD coating (a few min) yielded particles 30–100 nm in diameter; prolonged coating (over 1 h) produced micrometer-sized particles associated with color changes [45]. Our XPS results showed that the surface of the PCL scaffold exhibited characteristic peaks of C=O, C–O, and C–H, which are inherent to the PCL structure. After PD coating, the appearance of a C–N peak suggested successful surface modification by polydopamine. Our results suggest that an increase in the UV exposure time from 1 to 2 min enhances GelMA crosslinking, thus improving the GelMA storage modulus. For example, GelMA with varying degrees of functionalization (DoF) exhibited time-dependent proportional increases in the storage moduli; a higher DoF correlated with an elevated modulus [46].

### 3.3 Characterization of 3T3-L1 spheroids embedded in PCL scaffolds with the GelMA hydrogel

**Fig. 3:**
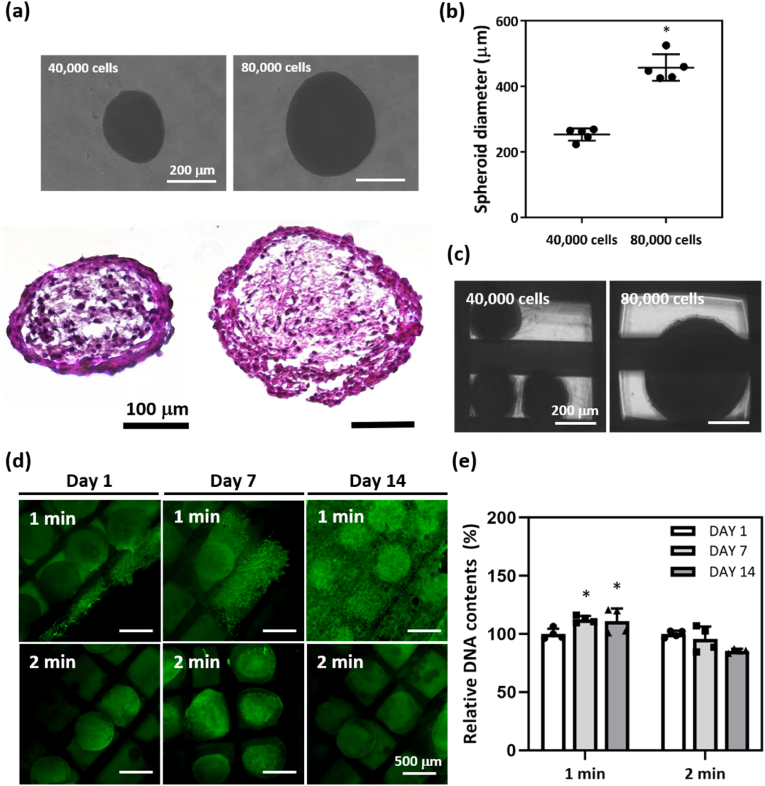
Characterization of 3T3-L1 spheroids embedded in a PCL scaffold with the GelMA hydrogel. (a) H&E-stained images of spheroids with 40,000 or 80,000 3T3-L1 cells. (b) Spheroid diameters (*n* = 5, independent experiments). (c) Phase-contrast images of a spheroid-laden PCL scaffold with a GelMA hydrogel containing 40,000 or 80,000 3T3-L1 cells. (d) Calcein-AM-stained images of a spheroid-laden GelMA hydrogel after 1 or 2 min of UV exposure taken on days 1, 7, and 14. (e) The relative DNA contents (*n* = 4, independent experiments) (∗p < 0.05).

The 3T3-L1 spheroids with 8 × 10^4^ cells that formed on Day 1 were larger than those with 4 × 10^4^ cells and exhibited a uniform cellular distribution on hematoxylin and eosin (H&E) staining in (Fig. 3a). Quantitatively, the diameter of spheroids with 8 × 10^4^ 3T3-L1 cells (457 ± 41 μm) was approximately 1.8-fold greater than that of those with 4 × 10^4^ 3T3-L1 cells (253 ± 19 μm) in (Fig. 3b). When spheroids with 4 × 10^4^ of 3T3-L1 cells were placed on the PCL scaffold, multiple spheroids occupied a single square space. In contrast, a spheroid with 8 × 10^4^ 3T3-L1 cells completely filled one space in (Fig. 3c). We observed adipose spheroids embedded in GelMA within PCL scaffolds for 30 days in Supporting Information (Fig. S7). Some spheroids lay in empty spaces of the PCL scaffold and, in the 1-min-UV-crosslinked GelMA hydrogel, 3T3-L1 cells of spheroids exhibited slight sprouting on Day 1. To Days 7 and 14, cells from the spheroids gradually filled the surrounding spaces. In contrast, 3T3-L1 cells in spheroids with hydrogel crosslinked for 2 min did not sprout (Fig. 3d). The relative DNA content of 3T3-L1 cells in spheroids within the 1-min-UV-crosslinked GelMA hydrogel gradually increased from Day 7 (112.59 ± 2.96 %) to Day 14 (111.14 ± 10.76 %) but decreased in hydrogel that was UV-exposed for 2 min from Day 7 (95.70 ± 10.76 %) to Day 14 (85.48 ± 1.69 %) in (Fig. 3e).

A volume of approximately 800 μ m within the PCL scaffold was optimal to obtain a uniform distribution of spheroids with 8 × 10^4^ 3T3-L1 cells. Spheroids formed via 3D stem cell self-assembly improved the differentiation capacity of the cells by maximizing cell–cell interactions and interactions between cells and the rich ECM [47]. Many investigations have focused on delivery of spheroids via scaffolds, emphasizing the critical need for precise size control [48,49]. Here, the cell sprouting and proliferation observed in the 1-min-cross-linked GelMA hydrogel is likely attributable to its weaker mechanical properties compared to those of the 2-min-cross-linked hydrogel. In recent decades, 3D tissue engineering involving cell encapsulation in hydrogels that mimic the physicochemical properties of natural tissues has received considerable attention [50]; the high tunability, hydrophilicity, biocompatibility, and degradability of hydrogels enable precise tissue formation [51]. Previous studies have indicated that substrate stiffness influences various cellular behaviors such as migration and sprouting [52]. For example, the filopodia of cells extended in all directions only in the softer of two GelMA hydrogels with strengths of 0.5 and 23 kPa, respectively [53]. We found that the mechanical properties of the hydrogel that underwent 1-min of UV exposure, in terms of encapsulating spheroids, were appropriate for recreation of the microenvironment within which 3T3-L1 cells sprouted, thus approximating the external matrix required for cell survival.

### 3.4 Adipogenesis in 3T3-L1 spheroid-laden PCL scaffolds with a GelMA hydrogel

**Fig. 4:**
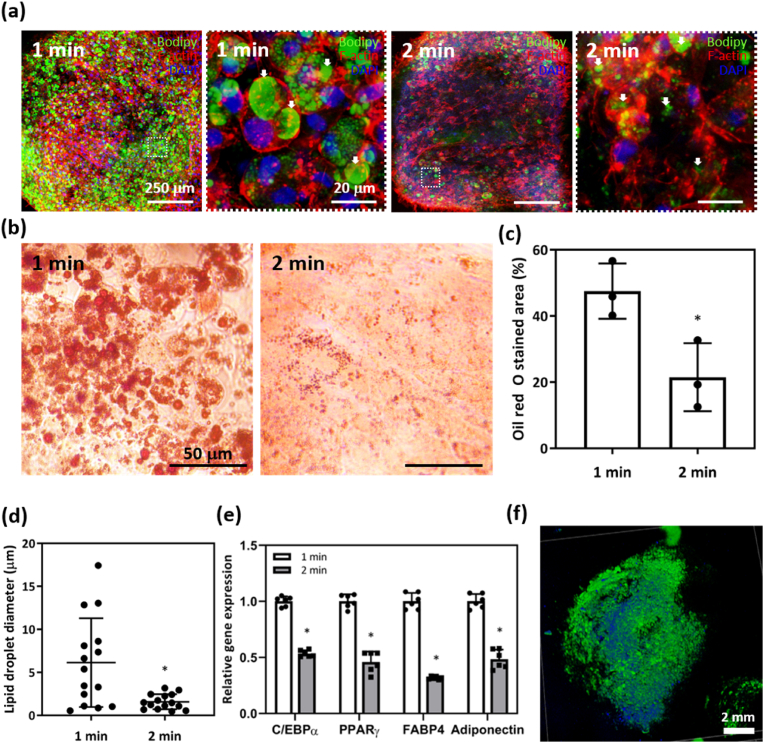
Adipogenesis within a 3T3-L1 spheroid-laden PCL scaffold with the GelMA hydrogel. (a) Confocal microscopic image of a 3T3-L1 spheroid within a PCL scaffold containing the GelMA hydrogel. White arrows indicate lipid droplets. (b) Phase-contrast images obtained after oil red O staining and (c) quantification of the stained area (*n* = 3, independent experiments). (d) Diameters of the lipid droplets (*n* = 15, samples; average per image view). (e) Relative gene expression levels of the adipogenic markers C/EBPα, PPARγ, FABP4, and adiponectin (*n* = 6, independent experiments). (f) Wide-view image of an adipose spheroid stained with BODIPY and DAPI (∗p < 0.05).

The BODIPY signals (green fluorescence) observed within the cytoplasm of cells in the 3T3-L1 spheroid-laden PCL scaffold with the 1-min-UV-crosslinked GelMA hydrogel were more frequent and notably larger compared to those within the 2-min-crosslinked hydrogel, from which the signals were relatively infrequent and smaller in (Fig. 4a). Additionally, after oil red O staining, red droplets were noticeably more widespread when the former hydrogel was used in (Fig. 4b). Quantitatively, the area covered by the latter hydrogel (21.50 ± 10.26 %) was significantly smaller compared to that of the former hydrogel (47.55 ± 8.35 %) in (Fig. 4c). The measured diameters of the observed droplets were significantly smaller in the latter (1.60 ± 0.80 μ m) than the former (5.08 ± 4.90 μ m) hydrogel in (Fig. 4d). The relative gene expression levels of adipogenic markers (C/EBP, PPAR γ, FABP4, and adiponectin) were significantly lower in the latter compared to the former hydrogel in (Fig. 4e). Entire spheroids were imaged using a confocal microscope in (Fig. 4f). We found that adipogenic differentiation of 3T3-L1 spheroids was influenced by the surrounding hydrogel microenvironment, as were proliferation and sprouting. We thus inferred that the mechanical properties of the hydrogel that was UV- cross-linked for 1 min were optimal in terms of cell differentiation. In previous works, the matrix stiffness was found to shape the cellular microenvironment and to regulate the differentiation of adherent cells, such as stem cells and pre-adipocytes, by influencing the expression of cytokines and growth factors [54]. Generally, networks of stiff hydrogels became denser upon cell spreading, negatively affecting cellular dynamics in terms of cytoskeletal organization, cell migration, and matrix metalloprotein secretion (and thus the hydrogel degradation rate) [55,56]. Tissue formation is possible only when signaling between cells and ECM molecules is appropriately regulated. Therefore, the mechanical properties of the hydrogel must allow for cell sprouting if 3D tissue engineering is to succeed [57,58]. Most prior studies investigated the effects of the mechanical hydrogel environment on encapsulated cells; the effects on encapsulated spheroids were seldom explored. In this study, the mechanical properties of the GelMA hydrogel were controlled by the UV irradiation time, and active cell spreading and differentiation were achieved within the hydrogel when the storage modulus was tuned to 4–500 Pa, similar to the results of a previous study [53].

### 3.5 GelMA physical properties

**Fig. 5:**
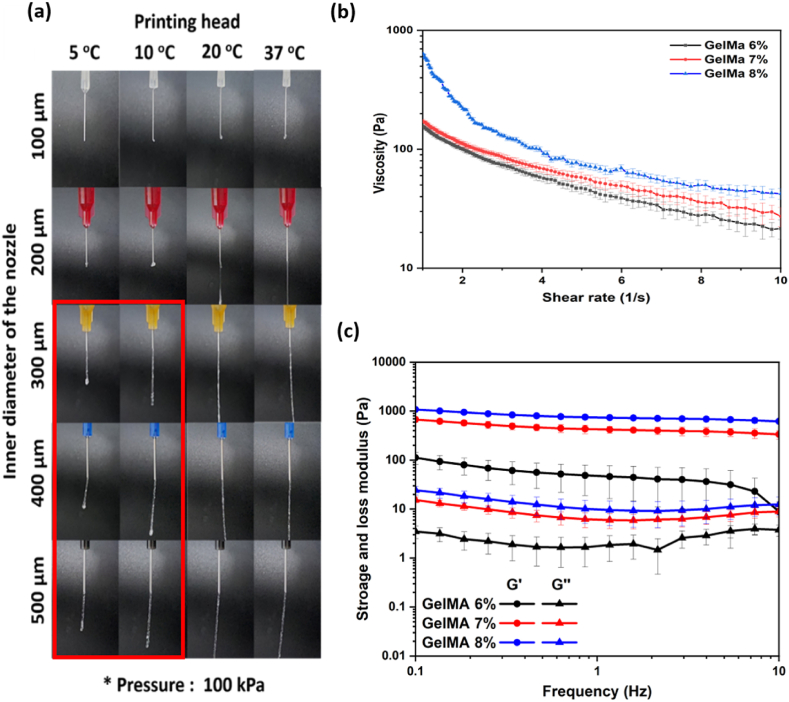
Characterization of the GelMA bioink used for 3D bioprinting. (a) Bioprinting extrudability depends on the head temperature and the nozzle inner diameter. (b) Rheological properties of the GelMA bioink. (c) Viscosity of the GelMA bioink.

We used nozzles of various diameters (100–500 μm) and a printing head, the temperature of which could be controlled between 5°C and 37°C. Before each printing extrusion test, the temperature of the area surrounding the outside of the syringe was held at 5 °C, 10 °C, 20 °C, or 37 °C for about 10 min. As the 100-μm nozzle was small, print extrusion was difficult. The 200-μm nozzle extruded when the external temperature of the syringe was 20 °C but printing was not possible because the ink was in solution. When using a 300–500-μm nozzle, if the external temperature of the syringe was >20 °C printing was not possible because the bioink was in solution, but printing became possible when the external temperature of the syringe was lowered to 10 °C. Printing below 10 °C was confirmed via detection of physical cross-linking (red square box in Fig. 5a). The pneumatic pressure was maintained at 100 kPa. When printing the skin layer, physical cross-linking proceeded for 10 min at an external syringe temperature of 10 °C using a 500-μm nozzle in (Fig. 5a). As physical cross-links established by controlling the temperature of the GelMA lack stability, we employed chemical bonding (UV cross-linking) to ensure that the skin layer would be maintained regardless of temperature. To investigate the viscoelastic physical properties of GelMA, we employed rheology, which directly reflects printability. At GelMA concentrations of 6 %, 7 %, and 8 %, the viscosity tended to decrease as the shear rate increased, and shear thinning, which becomes evident when printing is possible, was observed in (Fig. 5b) [59,60]. As the concentration of GelMA increased, the viscosity also rose, and the storage modulus G′ was higher than the loss modulus G″ in (Fig. 5c).

### 3.6 Characterization of skin layers with various GelMA concentrations and NIH3T3 fibroblasts, and co-culture with HUVECs

**Fig. 6:**
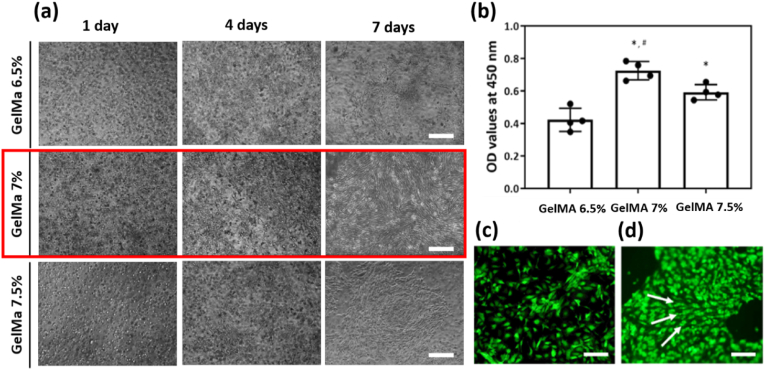
Analysis of NIH3T3 cell-laden skin layers with various GelMA concentrations for up to 7 days. (a) Microscopic images of NIH3T3 cells within a GelMA hydrogel embedded in a PCL scaffold. (b) Graph of the NIH3T3 cell proliferation rate according to the GelMA concentration. (c) Fluorescent image of GFP-HUVECs in 2D culture. Scale bar: 500 μm. (d) A fluorescent image of co-cultured NIH3T3 cells and GFP-HUVECs in 2D culture. Scale bar: 500 μm.

We observed (for 7 days) whether NIH3T3 fibroblast cells, which are skin cells, grew well at any GelMA concentration from 6.5 % to 7.5 %. It was microscopically confirmed that NIH3T3 cells grew best in 7 % GelMA in (Fig. 6a). On Day 7, the WST-1 test was used to determine cell proliferation, and it confirmed that the highest proliferation occurred in 7 % GelMA in (Fig. 6b). When cells proliferate, both proliferation and differentiation can be controlled by the surrounding environment [61,62]. To vascularize the skin layer, NIH3T3 fibroblasts were co-cultured with GFP-HUVECs, and we confirmed that GFP-HUVECs proliferated in three directions (white arrows) in (Fig. 6d). However, this was not observed when only GFP-HUVECs were cultured in (Fig. 6c), presumably because GFP-HUVECs were influenced by NIH3T3 fibroblast cells.

### 3.7 Effective vascularization of skin layers with fibroblasts and 3D microgel-coated HUVECs

**Fig. 7:**
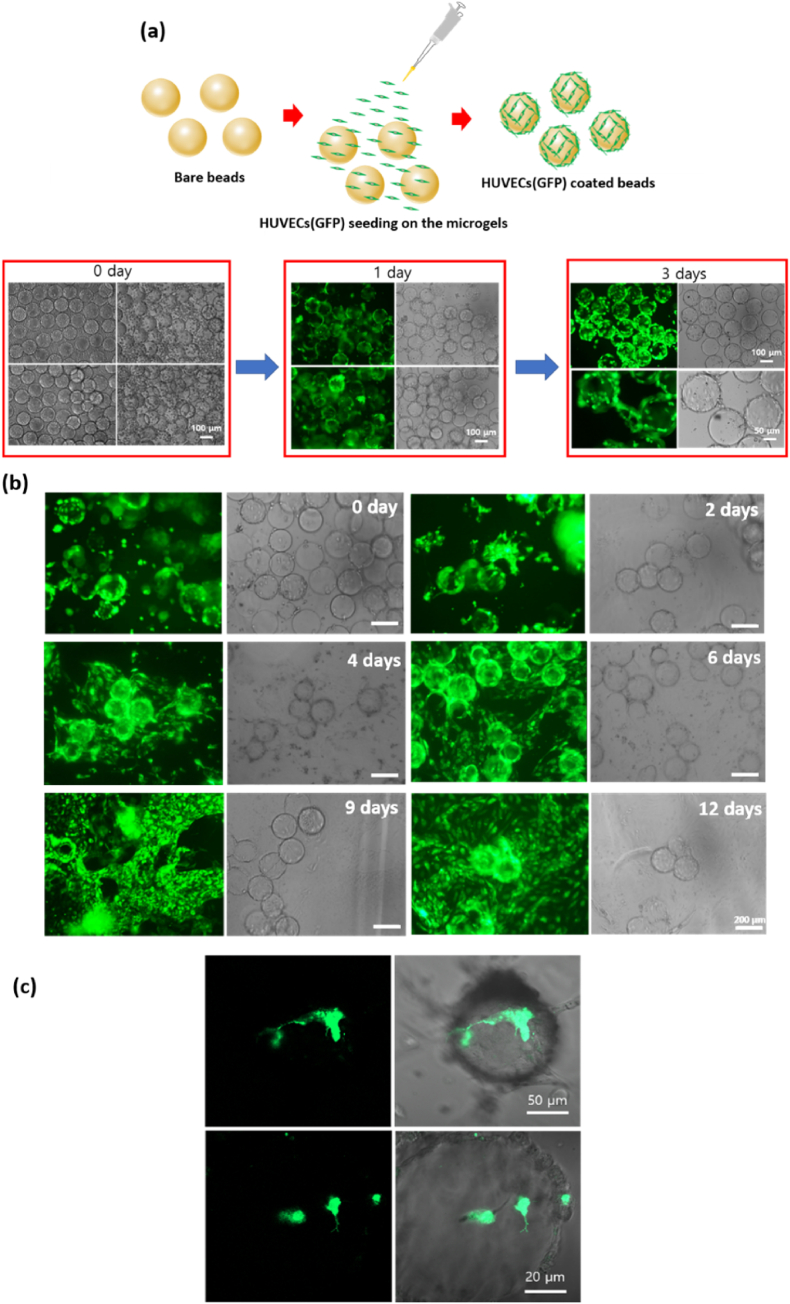
(a) Schematic of a microgel coated with GFP-HUVECs via direct seeding followed by culture for up to 3 days. Scale bar: 100 μm. (b) Fluorescent (left) and optical (right) microscopic images obtained over up to 12 days after transfer of the GFP-HUVECs-coated microgel to the GelMA surface (c) Fluorescent (left) images, and fluorescent images merged with optical (right) microscopic images, of the GFP-HUVECs-coated microgel surface confirmed de novo vascularization.

To effectively introduce blood vessels into skin layers composed of NIH3T3 fibroblasts, GFP-HUVECs were allowed to proliferate on the surface of a 3D microgel [63]. Microgels have a larger surface area than 2D cultures, enhance cell activity via stimulation by the surrounding environment, and model how different types of cells interact. GelMA is a biocompatible material, i.e., a gelatin-based polymer derived from collagen, and it contains the elements required for cell attachment via the functional amine group of the arginine-glycine-aspartic acid (RGD) peptide. Therefore, cell culture was possible on the surface of a microgel composed of GelMA [64,65]. Observations were conducted over 3 days; GFP-HUVECs became vascularized and proliferated on the surface of the microgel in (Fig. 7a). As GFP-HUVECs proliferating on the surface of a microgel must be vascularized within the skin layer, the spread of GFP-HUVECs on the surface of the GelMA microgel was observed for 12 days. GFP-HUVECs were observed not only around the microgel but also in areas lacking microgel, suggesting that the skin layer had become vascularized in (Fig. 7b). A confocal microscope was used to observe the areas around the microgel in detail; this confirmed the presence of new blood vessels in (Fig. 7c). We observed the appearance of blood vessel formation after transplanting microgels coated with HUVECs onto the GelMA surface. As a result, we were able to confirm the appearance of vascularization as HUVECs coated on the microgel spread in (Fig. S8).

### 3.8 Mimicking skin and adipose tissue

**Fig. 8:**
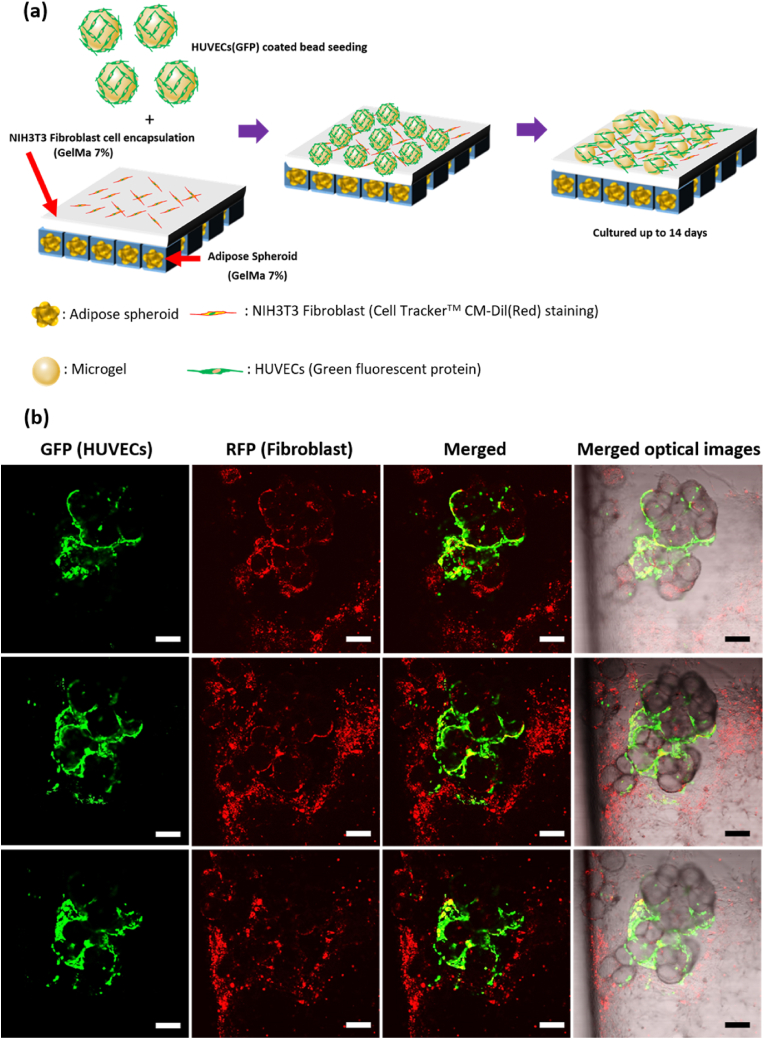
(a) Schematic of adipose/skin complex tissue with a GFP-HUVECs-coated microgel. NIH3T3 staining, and HUVECs detected by Cell Tracker™ CM-Dil (red) and HUVEC-specific GFP expression. (b) Complex tissues at different focal depths at the same position. Scale bar: 100 μm.

When printing skin layers onto PCL scaffolds with fat spheroids, we stained NIH3T3 fibroblasts with Cell Tracker™ CM-Dil (Red) to distinguish the fibroblasts from the GFP-HUVECs in the skin layer (Fig. 8a). shows a schematic of how we fabricated a composite tissue having a fat layer with fat spheroids and a skin layer using a microgel coated with GFP-HUVECs. As the microgel is 3D in nature and has a large surface area, GFP-HUVECs were well-cultured and proliferated effectively even when they were transferred to a skin layer composed of GelMA. When the same location was subjected to confocal microscopy at three different focal lengths, both the GFP-HUVECs coated onto the microgel surface and fibroblasts stained with Cell Tracker™ CM-Dil (Red) in the skin layer were observed in (Fig. 8b). The two types of cells were co-positioned in the skin layer; our *in vitro* model is thus more effective than earlier models [66]. We obtained the same results in other locations (Fig. S9).

There are some images from different positions. As explained earlier, you can see the presence of vascularization as well as the presence of dermal cells and epithelial cells.

### 3.9 Fabrication of dual: skin and adipose layers

**Fig. 9:**
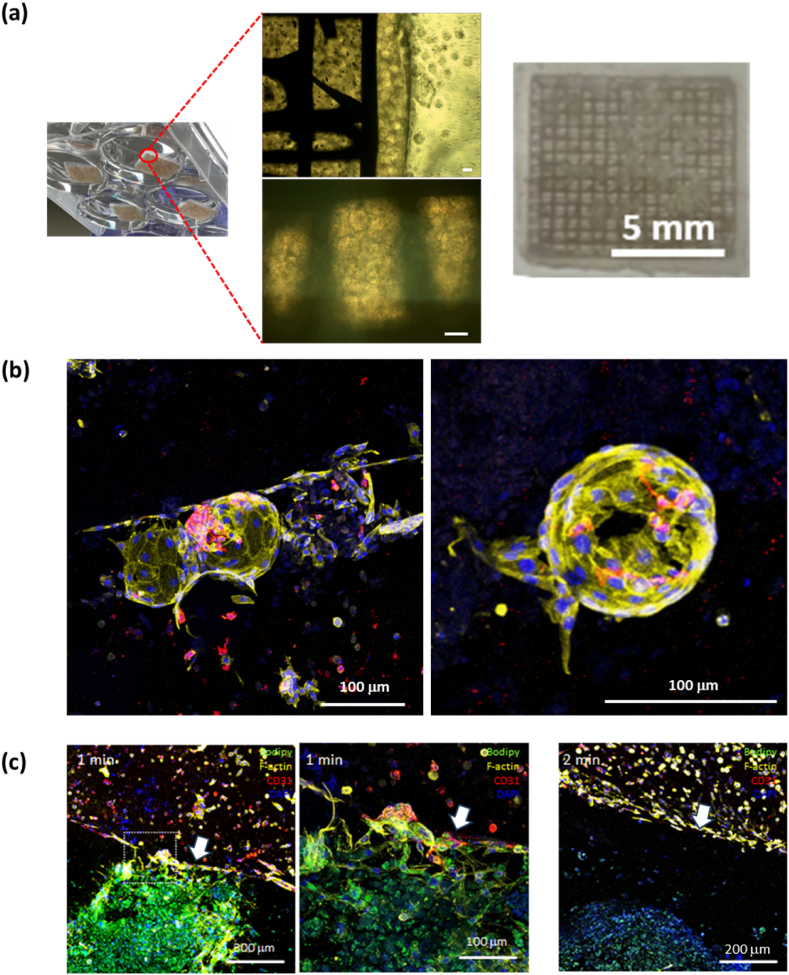
(a) Images of real adipose spheroid/skin complex tissues. Scale bar: 100 μm. (b) Confocal images of a GFP-HUVECs-coated microgel that was immunohistochemically stained. (c) Images of adipose/skin complex tissue revealed by immunohistochemical staining when the GelMA cross-linking time was 1 or 2 min. The white square indicates the interface of the two layers; a high-magnification image of the square is shown on the right. The white arrow indicates to the interface area.

After 5 days of culture, cells from both the skin and adipose layers came into contact because they migrated. The boundaries between the two layers disappeared after 10 days of culture; cells from each layer migrated into distinct areas within the 3T3-L1 spheroid-laden PCL scaffold with the GelMA hydrogel that had been crosslinked for 1 min under UV light. In contrast, after 2 min of cross-linking, the distinct interface between the two layers was preserved, and the cells did not migrate to either layer (Fig. 9b). The changes in the mechanical properties of the adipose layer, which depended on the duration of UV exposure, were intricately linked to the extent of interaction with the overlying skin layer. The fact that cell migration and proliferation were enhanced when hydrogel cross-linking proceeded for 1 rather than 2 min suggested that the dual-layer interactions were superior under the former condition. In previous studies, a hydrogel with chemically cross-linked DNA strands interconnected by silicate nanodisks (nSi) exhibited a high pore size and good plasticity, associated with marked protein release and notable migration of mesenchymal stem cells [67]. A 3D microenvironment, the mechanical properties of which are controlled, should in theory optimize the integration of dual layers. We found that such an environment played a crucial role in our successful development of an adipose and skin dual-layer system.

## 4 Conclusion

An adipose layer was generated using GelMA loaded with 3T3-L1 spheroids within a PCL scaffold, and a skin layer was created by printing GelMA containing fibroblasts and HUVECs on top of the adipose layer to create a bilayer adipose/skin composite tissue. 3T3-L1 cells were first grown as spheroids and then added to the fat layer. The GelMA strength was controlled by adjusting the UV cross-linking time. After cross-linking for 1 min, both the gene expression levels and the lipid droplet size were greater than after cross-linking for 2 min, and the staining range of oil red O was wider, suggesting differentiation into fat. To create a skin layer on top of a fat layer, GFP-HUVECs were attached/coated onto the surface of the microgel to effectively induce vascularization and then co-cultured with red-stained fibroblasts to create a skin layer with embedded blood vessels. As a microgel is 3D in nature, the surface area is large and vascularization thereof is more readily achieved than when a 2D microgel is employed. Analysis of the fat/skin composite tissue revealed interactions between cells of the skin and fat layers when cross-linking proceeded for 1 min, but not 2 min, because the physical properties of GelMA were optimal after cross-linking for only 1 min. We used 3D printing technology to create a platform for *in vitro* experiments that position cells at desired locations and employs a PCL scaffold supported by GelMA.

## CRediT authorship contribution statement

**Dongjin Lee:** Writing – original draft, Conceptualization. **Sangmin Lee:** Writing – original draft, Formal analysis. **Jeongbok Lee:** Data curation. **Dahong Kim:** Methodology. **Hyunseok Kwon:** Conceptualization, Formal analysis, Methodology. **Junhyoung Ahn:** Investigation. **Hyungjun Lim:** Conceptualization. **Jae Jong Lee:** Conceptualization. **Heungsoo Shin:** Writing – review & editing, Investigation. **Su A Park:** Writing – review & editing, Supervision, Funding acquisition, Conceptualization.

## Declaration of competing interest

The authors declare that they have no known competing financial interests or personal relationships that could have appeared to influence the work reported in this paper.

## Data Availability

No data was used for the research described in the article.
