# Supplementary material for: Vascularized skin tissue models featuring adipose cell spheroid-laden GelMA hydrogels
Source: Mater Today Bio. 2025 May 5;32:101835. doi: 10.1016/j.mtbio.2025.101835 (PMC12136904; doi:10.1016/j.mtbio.2025.101835)
Supplement: Multimedia component 1 [file mmc1.docx]

**Supplementary Figures**

**
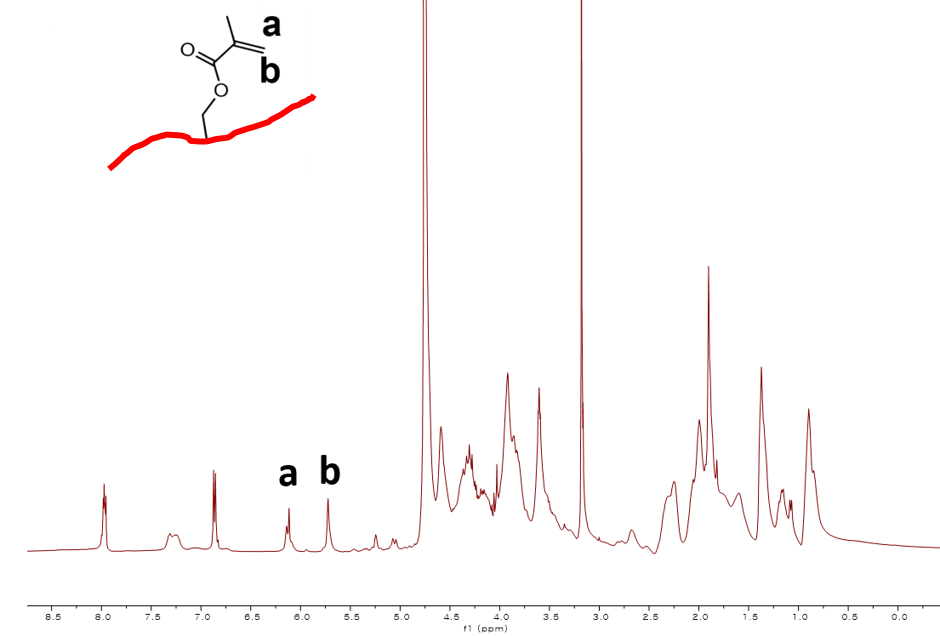
**

Figure S1. ^1^H-NMR spectrum of the GelMA hydrogel. (a and b) The two methacrylic peaks.


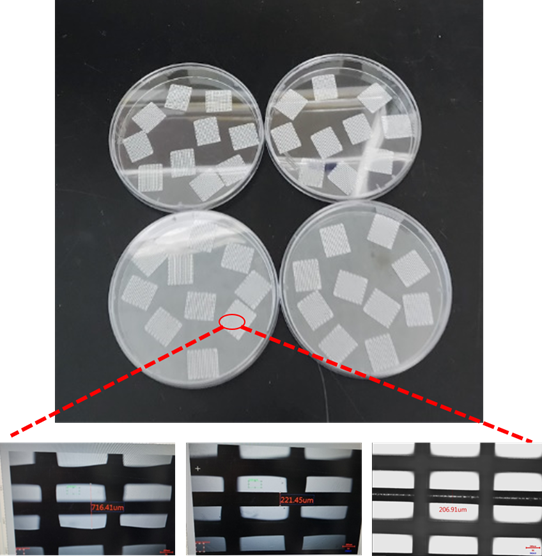


Figure S2. PCL scaffold support that forms the adipose tissue layer.

**
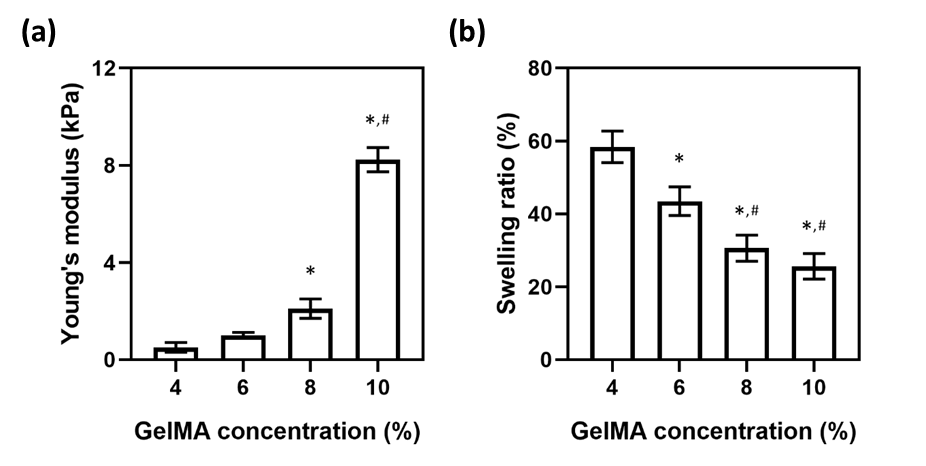
**

Figure S3. Elastic moduli and swelling ratios associated with various concentrations of GelMA.

**
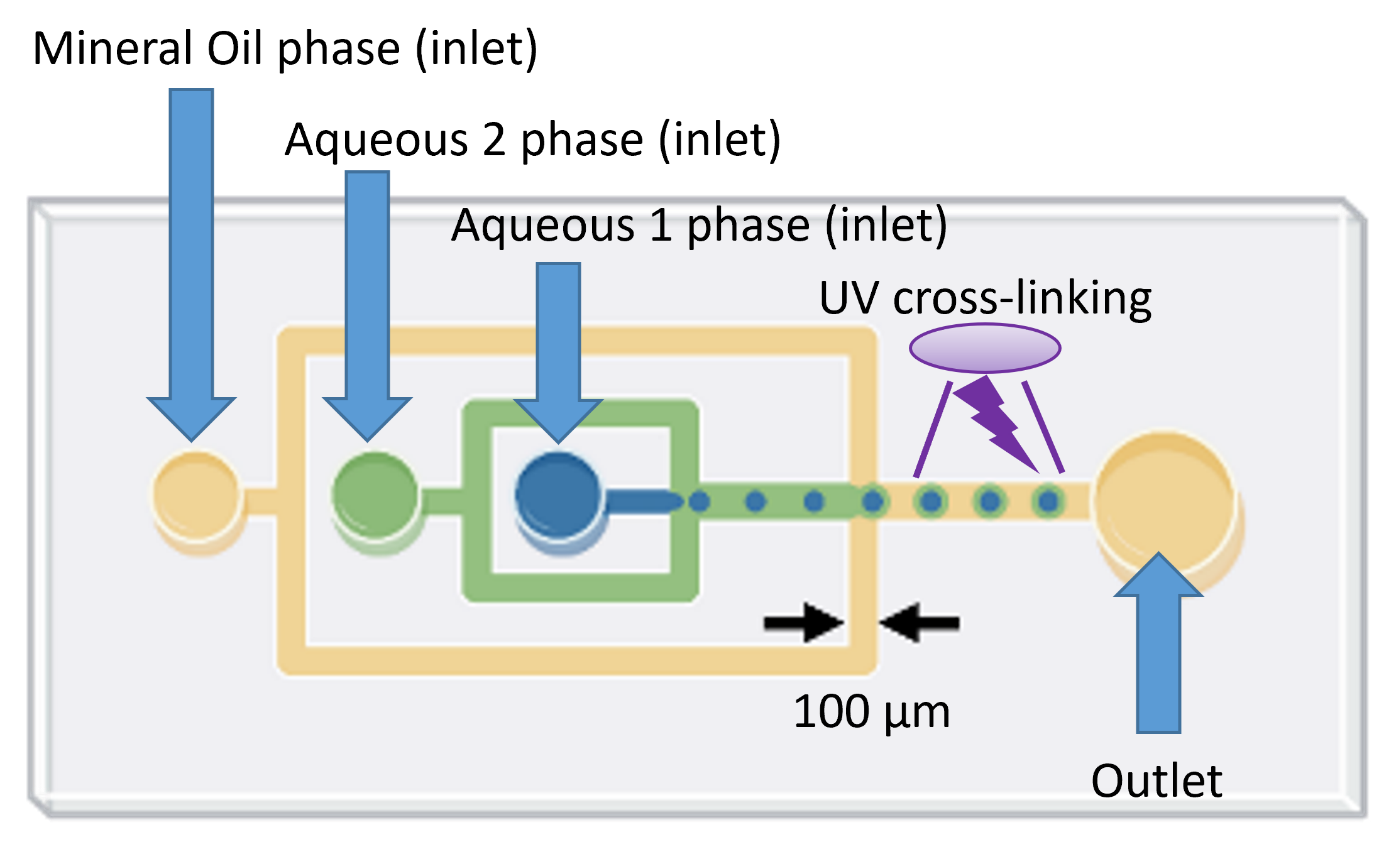
**

Figure S4. Channel geometry of the microfluidic device. Droplets were converted into microgels via UV cross-linking.


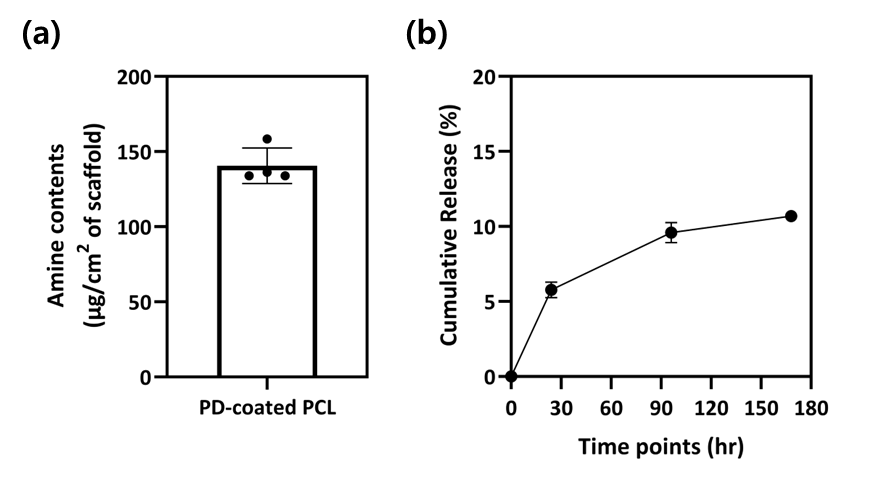


Figure S5. (a) Quantification of catecholamine coated on PD-coated PCL scaffold. (b) Cumulative release of catecholamine over 7 days. Quantification was performed using the Micro-BCA assay (*n* = 4 samples).


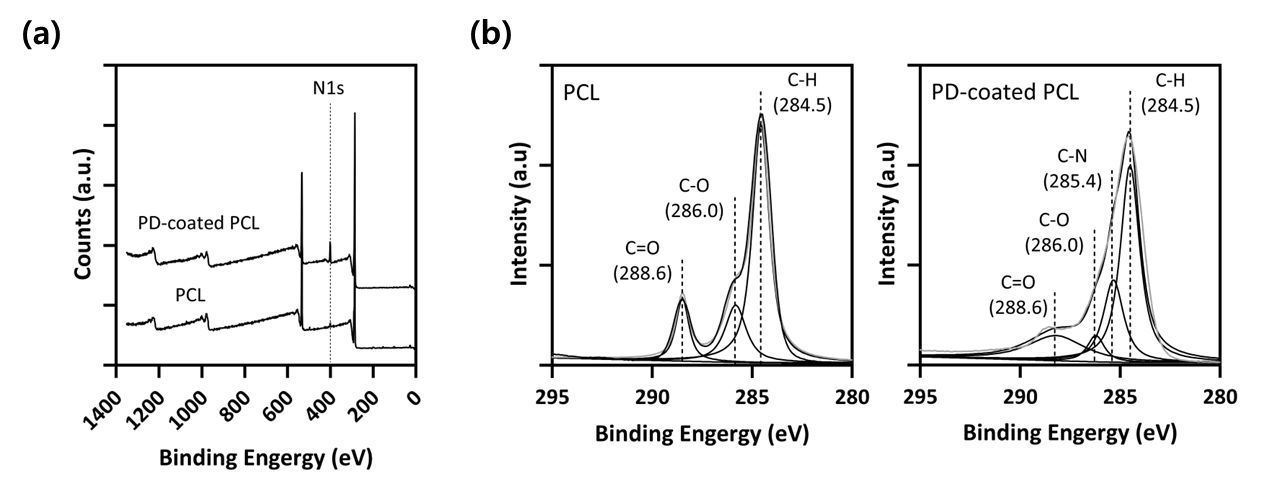


Figure S6. (a) Full spectra of XPS analysis and (b) high-resolution C1s spectra for PCL and PD-coated PCL scaffolds.

**
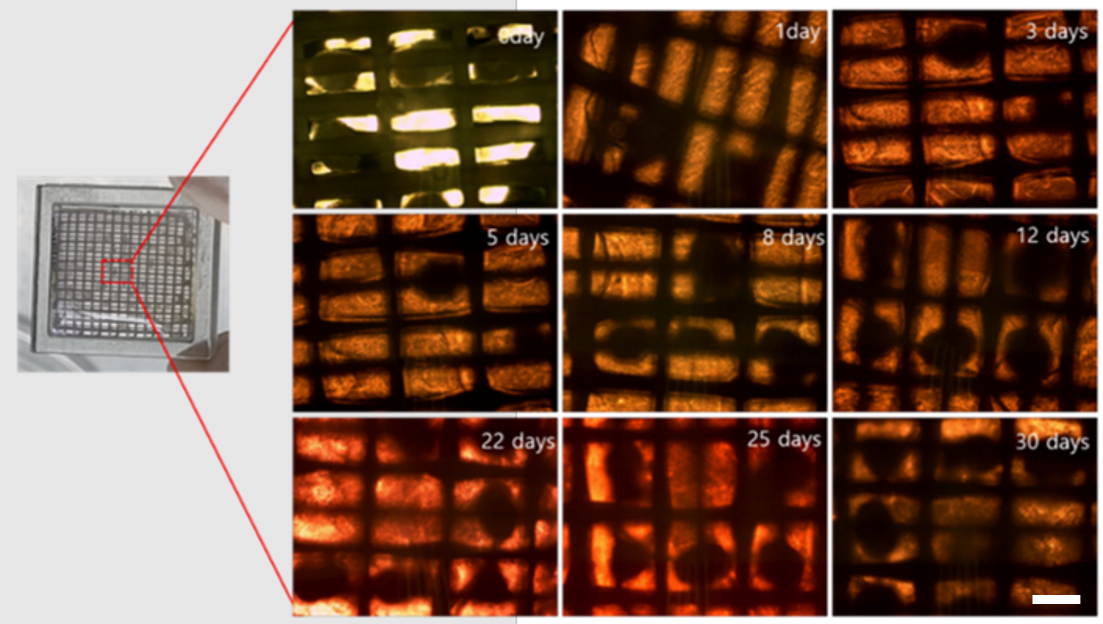
**

Figure S7. Long-term culture of adipose spheroids in a PCL scaffold filled with GelMA hydrogel for up to 30 days. Scale bar: 500 µm.

**
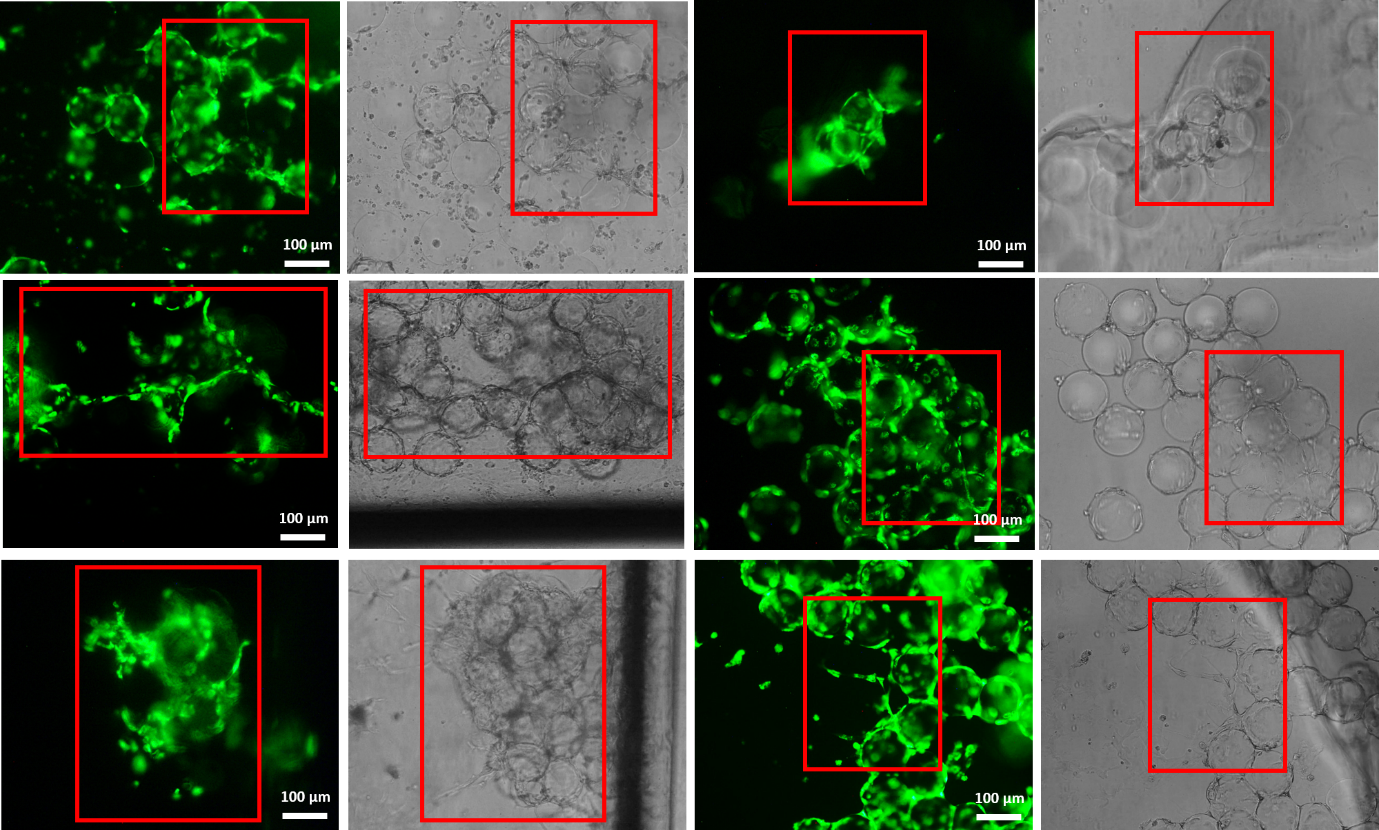
**

Figure S8. Appearance of vascularization after grafting a microgel coated with HUVECs onto the surface of GelMA. Scale bar: 100 μm.

**
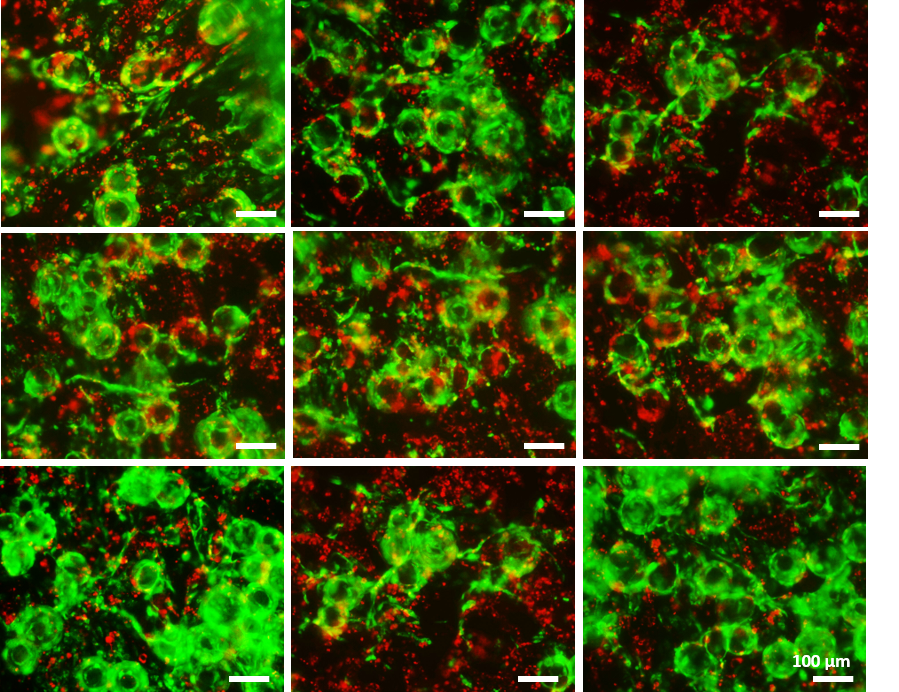
**

Figure S9. NIH3T3 staining, and HUVECs detected by Cell Tracker^TM^ CM-Dil (Red) and HUVEC-specific GFP expression. Appearance of vascularization after grafting a microgel coated with HUVECs in skin tissue. Scale bar: 100 μm.
